# Next-Generation Sequencing in Korean Children With Autism Spectrum Disorder and Comorbid Epilepsy

**DOI:** 10.3389/fphar.2020.00585

**Published:** 2020-05-14

**Authors:** Junghan Lee, Sungji Ha, Seung-Tae Lee, Sung-Gyun Park, Saeam Shin, Jong Rak Choi, Keun-Ah Cheon

**Affiliations:** ^1^Division of Child and Adolescent Psychiatry, Department of Psychiatry, Severance Hospital, Institute of Behavioral Science in Medicine, Yonsei University College of Medicine, Seoul, South Korea; ^2^Department of Psychiatry, Institute of Behavioral Science in Medicine, Yonsei University College of Medicine, Seoul, South Korea; ^3^Department of Laboratory Medicine, Yonsei University College of Medicine, Seoul, South Korea

**Keywords:** autism spectrum disorder, epilepsy, next-generation sequencing, clinical exome sequencing, autism genetics

## Abstract

Autism spectrum disorder (ASD) is a neurodevelopmental disorder characterized by impairments in social communication and restricted and repetitive behaviors and interests. Identifying the genetic background may be one of the key features for the future diagnosis and treatment of ASD. With the tremendous development in genetic diagnosis techniques, next-generation sequencing (NGS) can be used to analyze multiple genes simultaneously with a single test in laboratory and clinical settings and is well suited for investigating autism genetics. According to previous studies, there are two types of genetic variants in ASD, rare variants and common variants, and both are important in explaining pathogenesis. In this study, NGS data from 137 participants with ASD were reviewed retrospectively with consideration for comorbid epilepsy. Diagnostic yield was 17.51% (24/137), and pathogenic/likely pathogenic variants were seen more frequently in female participants. Fourteen participants were diagnosed with comorbid epilepsy, six of them had pathogenic/likely pathogenic variants (43%). Genes with variants of unknown significance (VOUS) which have one or more evidence of pathogenicity following the American College of Medical Genetics (ACMG) criteria were also reviewed in both ASD and ASD with comorbid epilepsy groups. We found that most frequently found VOUS genes have previously been reported as genes related to ASD or other developmental disorders. These results suggest that when interpreting the NGS results in the clinical setting, careful observation of VOUS with some pathological evidence might contribute to the discovery of genetic pathogenesis of neurodevelopmental disorders such as ASD and epilepsy.

## Introduction

Autism spectrum disorder (ASD) is a neurodevelopmental disorder with core symptoms of persistent deficits in social communication and restricted, repetitive patterns of behavior, interests, or activities (APA, 2013). According to recent reports, ASD no longer seems to be a rare disease; the overall prevalence is 16.8 per 1,000 children aged 8 years, with overall male-to-female ratio of 4:1 in the United States (Baio et al., 2018). Autism spectrum disorder is not simply a disorder but a significant social problem because the annual costs for ASD patients are tremendous (Lavelle et al., 2014). Costs may include medical and nonmedical costs and indirect costs such as parental productivity loss (Buescher et al., 2014). Accurate diagnosis and treatment guidance might substantially impact treatment of the disorder and reduce annual costs.

Autism spectrum disorder is associated with various coexisting factors including those related to genetics, the prenatal environmental, and the postnatal environmental (Lord et al., 2018). While knowledge about the neurobiological basis of ASD is still insufficient, genetic factors are regarded as crucial components according to previous studies (Rosenberg et al., 2009; Hallmayer et al., 2011). Studies of monozygotic twin concordance and sibling recurrence rates clearly reveal that genetic factors play important roles in the development of ASD (Geschwind, 2011; Sandin et al., 2014; Tick et al., 2016). In this context, identifying the genetic background of each ASD patient could be the ‘cornerstone’ of proper diagnosis and individualized treatment. In general, genetic diagnosis has the benefits of informing prognoses and preventing further superfluous invasive testing, leading to tailored treatment and family counseling (Han et al., 2018). In particular, genetic testing may be a key component in the development of precision medicine, in hope of predicting treatment outcomes on an individual basis. Tools for genetic analyses are rapidly developing and the collection of genetic information is accelerating tremendously (Geschwind and State, 2015; Loth et al., 2016).

However, there are inevitable obstacles to defining a genetic basis of ASD. First, the majority of ASD cases cannot be explained by a single gene mutation. Previous reports have indicated that only 10% of ASD cases originate from a rare variant in a single gene (Persico and Napolioni, 2013). Moreover, ASD can occur as a result of a combination of common variants (Gaugler et al., 2014). The fact that common variants are not causal for disease makes it harder for researchers to define their clinical importance (Geschwind, 2011; Persico and Napolioni, 2013). Second, several characteristics of autism genetics, such as an extremely heterogeneous genetic contribution, many different loci underlying disease, variable phenotypic expression, and lack of specificity make it difficult to understand the neuropathology of the condition (Persico and Napolioni, 2013). Solving heterogeneity may be the most important future task for ASD researchers.

It is clear that traditional candidate gene studies are not suited to investigate common gene variants. As next-generation sequencing (NGS) technologies developed, whole exome sequencing (WES) was used to identify diverse genetic variants, including common variants in ASD (Sanders et al., 2012; Persico and Napolioni, 2013). The use of NGS, with its ability to simultaneously analyze multiple genes (in case of WES) in a single test, is currently being established in clinics and laboratories (Yohe and Thyagarajan, 2017).

From a genetic perspective, an ASD patient should be monitored for comorbid epilepsy because ASD and epilepsy are known to share genetic backgrounds, which may be related to neuropathophysiology during brain development (Tuchman and Rapin, 2002; Tuchman and Cuccaro, 2011). Autism spectrum disorder patients with rare gene variants related to genetic syndromes such as Rett’s syndrome, are strongly suspected to have comorbid epilepsy (Canitano, 2007). Previous studies show that epilepsy in ASD is highly related to intellectual disability (ID) (Amiet et al., 2008) and associated with severity of ASD (Ko et al., 2016). To reveal the genetic background of neurodevelopment, examining the discriminative characteristics of genetic components of ASD with epilepsy and ID and comparing them with ASD with no comorbidities is essential. To investigate genetic variants associated with ASD and ASD with comorbid epilepsy, we planned a retrospective review of the medical records and NGS data of ASD patients.

## Materials and Methods

### Participants

We reviewed medical records of ASD patients who underwent NGS for genetic evaluation, who visited a specialist in an out-patient clinic for autism at the Severance Children’s Hospital, from January 1, 2016. In clinical settings, we recommend NGS to parents when patients show severe autistic symptoms, morphological problems, or other medical or neurological comorbidities. Data from 141 patients were collected, and four patients among them were excluded due to lack of clinical assessment and follow-ups which are vital for diagnosing ASD. One hundred and thirty-seven enrolled participants were clinically diagnosed as ASD by a specialized psychiatrist on the basis of diagnostic criteria suggested in DSM-5, and several clinical assessments (see *Clinical Assessments*) support the diagnosis. As data were reviewed retrospectively and it was impossible to fulfill assessments not performed in the clinical setting, there were missing scores for Intelligence-Quotient (IQ), the Social Responsiveness Scale (SRS), and the Social Communication Questionnaire (SCQ), in several participants. We examined not only NGS and clinical assessment data, but also checked for comorbid epilepsy and other comorbidities, history of seizure, and electroencephalogram (EEG) reports. For the cases with comorbid epilepsy, we followed the diagnostic decision of neurologists in the Severance Children’s Hospital. This study was approved by the applicable institutional Review Boards for research with human subjects at Severance Hospital, Yonsei University College of Medicine, where this study was performed.

### Clinical Assessments

All participants had a previous clinical diagnosis of ASD by a specialized child psychiatrist. The diagnosis of ASD was established using Autism Diagnostic Interview-Revised (ADI-R) and Autism Diagnostic Observation Schedule (ADOS), the gold standard for ASD diagnosis. Clinical autistic characteristics of the participants were supplemented by the CARS, SRS and SCQ.

The Childhood Autism Rating Scale (CARS) is a 15-item behavioral rating scale, developed to distinguish ASD and other developmental disorders and to assess the severity of ASD. Each item is scored from one to four points, and midpoint scores are also possible. Higher scores indicate more severe ASD symptoms. The cut-off score, which distinguishes ASD from other developmental disorders, is 30 points (Amiet et al., 2008). The reliability and validity of the Korean version of CARS (K-CARS) have been verified (Shin and Kim, 1998).

The SRS is a 65-item questionnaire that asks parents and/or teachers about the characteristics of the social interactions shown by children over the past 6 months (Constantino et al., 2000). Each question is scored from zero to three points, depending on the frequency of the action described in each item. Higher scores mean a lower social function. We previously confirmed the clinical validity of the SRS in Korean children and provided the Korean T-score norm (Cheon et al., 2016). In the present study, we used total T-score of the participants.

The SCQ is a 40-item screening instrument that is based on ADI-R, a tool for more in-depth assessment of ASD symptoms, and selects key items that deviate from normal development (Chandler et al., 2007). The Korean version of the Social Communication Questionnaire (K-SCQ) was verified as a reliable and valid instrument for screening autistic symptoms in the Korean population (Kim et al., 2015). Each question is answered with yes or no. Higher scores indicate more severe symptoms associated with ASD.

The ADI-R is a semi-structured parent interview tool for parents of children aged 2 years and older (Lord et al., 1994). This is generally conducted in conjunction with ADOS, which directly monitors and assesses the child, and is used to complement the interpretation of results (Lord et al., 2000).

To assess the cognitive levels of participants, we used the Korean-Wechsler Intelligence Scale for Children-IV (K-WISC-IV) and the Korean Wechsler Preschool and Primary Scales of Intelligence-IV (K-WPPSI-IV). We also used Korean-Bayley-III for children who were unable to perform the Wechsler’s intelligence scales because of their age or development status.

### Next-Generation Sequencing

The xGen Inherited Diseases Panel (Integrated DNA Technologies, Coralville, IA, USA) including 4,503 candidate genes was used for exome sequencing. Genes associated with various neurodevelopmental disorders such as ASD, epilepsy, seizure disorder, and X-linked ID are included in this panel.

Genomic DNA extracted from individuals’ samples was used for library preparation and target capture using a custom panel targeting candidate genes. Massively parallel sequencing was performed using the NextSeq 550Dx System (Illumina, San Diego, CA, USA). Quality control and sequence analysis was carried out using our custom analysis pipeline. Copy number analysis was carried out using our custom analysis pipeline (Kim et al., 2019). The GRCh37 (hg19) build was used as the reference sequence for mapping and variant calling while using Burrows-Wheeler alignment (BWA) tool (version 0.7.12). HaplotypeCaller and MuTect2 in the GATK package (3.8-0) and VarScan2 (2.4.0) were used to identify single nucleotide variations (SNV) and insertion and deletions (indels). Databases used for analyses and variant annotation include Online Mendelian Inheritance in Man (OMIM), the Human Gene Mutation Database (HGMD), Clinvar, dbSNP, 1000 Genomes, the Exome Aggregation Consortium (ExAC), the Exome Sequencing Project (ESP), and the Korean Reference Genome Database (KRGDB). Classification of variants followed the standards and guidelines established by the American College of Medical Genetics (ACMG) (Richards et al., 2015), with a scoring algorithm implemented in the DxSeq Analyzer (Dxome, Seoul, Korea). All pathogenic and likely pathogenic variants were further confirmed by Sanger sequencing.

Genetic variants that are not met for pathogenic/likely pathogenic nor benign/likely benign are classified as variants of unknown/uncertain significance (VOUS) according to the ACMG guideline. Benign and likely benign variants were excluded in our NGS clinical reports. If VOUS had one or more evidence of pathogenicity but unmet criteria for pathogenic/likely pathogenic, they were regarded as VOUS with a relatively high probability of pathogenicity. The VOUS with high probability of pathogenicity were selected by physicians in laboratory medicine referencing the criteria on evidence of pathogenicity in the ACMG guideline. Among VOUS with high probability of pathogenicity, we selected five or less variants for analysis.

### Statistics

To compare demographic characteristics and results of clinical assessments between patients with and those without pathogenic/likely pathogenic variants, we used the Chi-squared test and the independent t-test. Statistical significance was defined at *p* < 0.05. Analyses were performed using the Statistical Package for the Social Sciences software (version 25.0; SPSS Inc., Chicago, IL, USA).

## Results

Among 137 patients, only three patients showed no pathogenic/likely pathogenic variants nor VOUS according to our NGS clinical reports. Seven cases were identified with pathogenic variants, and 17 participants had likely pathogenic variants. The diagnostic yield acquired from the total NGS data was about 17.51% (24/137). Differences in demographic information and clinical assessment results are presented in **Table 1**. The proportion of females to males was significantly higher in the pathogenic/likely pathogenic variants group (62.5%, *p* = 0.006). The pathogenic/likely pathogenic variants group was associated with higher incidence of comorbid epilepsy (25%, *p* = 0.008). There were no between-group differences in age and clinical assessment scores (IQ, SRS T-score, SCQ, CARS). Characteristics of epilepsy and reports of electroencephalogram (EEG) in ASD with comorbid epilepsy were listed in **Supplementary Material 1**.

**Table 1:** Demographic data of participants.

	Patients with pathogenic/likely pathogenic variants (n = 24)	Patients without pathogenic/likely pathogenic variants (n = 113)	*p*-value	Total (n = 137)
**Male: Female (female/male ratio, %)**	9:15 (62.5%)	76:37 (32.7%)	**0.006**	85:52
**Age (months)**	65.21	60.58	0.463	61.39
**IQ**	50.88	53.51	0.395	53.02
**SRS (total T-score)**	86.79	86.29	0.933	86.39
**SCQ**	17.67	16.82	0.639	16.99
**CARS**	33.184	31.669	0.402	31.935
**Comorbid ID**	20 (83.3%)	84 (74.3%)	0.349	104 (75.9%)
**Comorbid epilepsy**	6 (25%)	8 (7.1%)	**0.008**	14 (10.2%)

Pathogenic/likely pathogenic variants appeared more frequently in the female group (62.5%) than in the male group (p-value = 0.006). There were no remarkable differences in age and clinical assessments (IQ, SRS, SCQ, CARS) between the two groups. Comorbid intellectual disability was prominent in both groups, while comorbid epilepsy was more frequently diagnosed in pathogenic/likely pathogenic variants group (p = 0.008). IQ, Intelligence quotient; SCQ, Social Communication Questionnaire; CARS, The Childhood Autism Rating Scales; ID, Intellectual disability. **BOLD: p < 0.05**.

By comparing males with females (**Table 2**), we found that females appear to have higher scores for the SRS total T-score (*p* = 0.024) as well as frequently detected pathogenic/likely pathogenic variants (*p* = 0.006). The CARS score was also slightly higher in females (*p* = 0.045), while age and other scores (IQ, SCQ) showed no significant statistical differences.

**Table 2:** Male–Female comparison.

	Male	Female	*p*-value
**Pathogenic/likely pathogenic variants**	9 (10.6%)	15 (28.8%)	**0.006**
**Age**	61.93	60.52	0.792
**IQ**	53.48	52.24	0.616
**SRS (total T-score)**	82.86	93.91	**0.024**
**SCQ**	16.58	17.75	0.434
**CARS**	31.16	33.25	**0.045**
**Comorbid ID**	65 (76.5%)	39 (75.0%)	0.845
**Comorbid epilepsy**	6 (7.1%)	8 (15.4%)	0.118

There were more females who have pathogenic/likely pathogenic variants (p = 0.006). Females showed higher SRS T-score (p = 0.024) and CARS score (p = 0.045) on average. There were no significant differences in age, IQ, SCQ, and comorbidity of ID, epilepsy. IQ, Intelligence quotient; SCQ, Social Communication Questionnaire; CARS, The Childhood Autism Rating Scales; ID, Intellectual disability. **BOLD: p < 0.05**.

Genes that harbored pathogenic variants included *SHANK3, PTEN, NSD1, PAFAH1B1*, and *RAI1*. Mutation types include exon deletion and nonsense mutations. We also identified copy number variants (CNV), chromosome 8p23.2 duplication, and chromosome 15q11.2–q13.2 duplication. These variants were expected to lead to loss of genetic function (Richards et al., 2015) which may play a role in pathogenesis of disease. Genetic information from OMIM were also described in **Table 3**. Among pathogenic variants, only *PAFAH1B1* was not previously reported to be related to neurodevelopmental disorder including ASD, ID, and epilepsy.

**Table 3:** Genetic characteristics: genes with pathogenic variants.

No.	Gene	Accession	Nucleotide	Amino acid	Diseases (OMIM)	Zygosity	Inheritance^a^ (OMIM)	ACMG
5	*SHANK3*		Deletion (exon 9–22)		**Phelan–McDermid syndrome** {Schizophrenia 15}	Hetero		
6	**8p23.2 duplication (2.25 Mb)							
16	*PAFAH1B1 (LIS1)	NM_000430.3	Exon 4 deletion		Lissencephaly 1; Subcortical laminar heterotopia	Hetero	AD	
17	*RAI1*	NM_030665.3	Exon 6 deletion		**Smith–Magenis syndrome**	Hetero	AD	
46	*PTEN*	NM_000314.4	c.249C > A	p.Cys83Ter	**Cowden syndrome 1** **Macrocephaly/autism syndrome** Bannayan**–**Riley**–**Ruvalcaba syndrome; Endometrial carcinoma, somatic; {Glioma susceptibility 2}; Lhermitte**–**Duclos syndrome; Malignant melanoma, somatic; {Meningioma}; PTEN hamartoma tumor syndrome; {Prostate cancer, somatic}; Squamous cell carcinoma, head and neck, somatic; VATER association with macrocephaly and ventriculomegaly	Hetero	AD,AR	PVS1, PM2, PM6
68	15q11.2q13.2 duplication (9.5Mbp)							
136	*NSD1*	NM_022455.4	c.6349C > T	p.Arg2117Ter	**Sotos syndrome 1** Beckwith**–**Wiedemann syndrome; Leukemia, acute myeloid	Hetero	AD	PVS1, PM2, PP5

Pathogenic variants that were found in seven participants. OMIM, Online Mendelian Inheritance in Man; ExAC, population frequency from The Exome Aggregation Consortium; KRGDB, population frequency from the Korean Reference Genome Database; AD, Autosomal dominant; AR,Autosomal recessive; XD, X-linked dominant; XR, X-linked recessive; ACMG, The American College of Medical Genetics and Genomics guideline (Richards et al., 2015); PVS, Very strong evidence of pathogenicity; PM, Moderate evidence of pathogenicity; PP, Supporting evidence of pathogenicity. ^a^Inheritance of the gene described in OMIM. *not previously reported to be associated with neurodevelopmental disorders (ASD, ID, epilepsy). **genetic variants in ASD with comorbid epilepsy. **BOLD: Clinical syndromes and diseases related to neurodevelopmental disorders (ASD, ID, epilepsy)**.

Likely pathogenic variants showed various types of mutations such as copy number variants, exon deletion, nonsense mutation and missense mutation (**Table 4**). Both patients with variants in *TSC2* were diagnosed with tuberous sclerosis clinically. Likewise, both patients with variants in *MECP2* were diagnosed with Rett’s syndrome in clinical setting. While most of genes are known to be related to ASD, ID or epilepsy, *ABCC2, CCDC50* and *SLC26A4* were not reported to be related to neurodevelopmental disorder according to OMIM. Likely pathogenic group showed significantly lower SRS T-score compared to pathogenic group (**Supplementary Material 2**).

**Table 4:** Genetic characteristics: genes with likely pathogenic variants.

No.	Gene	Accession	Nucleotide	Amino acid	Diseases (OMIM)	Zygosity	Global frequency (ExAC)	Korean frequency (KRGDB)	Inheritance^a^ (OMIM)	ACMG
39	*ABCC2	NM_000392.3	c.2443C > T	p.Arg815Ter	Dubin**–**Johnson syndrome	Hetero	0.00002826		AR	PVS1, PM2
	*ABCC2	NM_000392.3	c.2302C > T	p.Arg768Trp	Dubin**–**Johnson syndrome	Hetero	0.00007539	0.000803859	AR	PP3
57	*MECP2*	NM_004992.3	c.403A > G	p.Lys135Glu	**{Autism susceptibility, X-linked 3}** **Mental retardation** **Rett syndrome** Encephalopathy, neonatal severe	Hetero			XR, XD	PM2, PP3, PP5
60	**Xp22.2p22.33 deletion					Hetero				
	**NLGN4X	NM_020742.3	Whole gene deletion		**Asperger syndrome susceptibility** **Autism susceptibility** **Mental retardation**	Hetero				
63	Xp22.31p22.33 deletion					Hetero				
	*NLGN4X*	NM_020742.3	Whole gene deletion		**Asperger syndrome susceptibility** **Autism susceptibility** **Mental retardation**	Hetero				
66	*DLGAP2*	NM_004745.4	Whole gene duplication		**Autism spectrum disorder**	Hetero				
69	*AUTS2*	NM_015570.2	c.2962dleG	p.Glu988LysfsTer37	**AUTS2 syndrome Mental retardation**	Hetero			AD	PVS1, PM2
75	*SCN2A*		Exon 15-16 deletion		**Epileptic encephalopathy, early infantile**	Hetero			AD	
76	*KAT6A*	NM_006766.3	c.3456G > A	p.Trp1152Ter	**Mental retardation**	Hetero				PVS1, PM2
	*CCDC50	NM_178335.2	c.82_83dupAC	p.Leu29ProsfsTer40	?Deafness,	Hetero			AD	PVS1, PM2
87	*HUWE1*	NM_031407.5	c.693+1G > A		**Mental retardation, syndromic, Turner type**	Hetero				PVS1, PM2
94	**TSC2	NM_000548.3	c.4744_4746del	p.Ile1582dle	**Tuberous sclerosis-2** Lymphangioleiomyomatosis, somatic	Hetero			AD	PM2, PM4, PM6
95	**TSC2	NM_000548.3	c.2838_122G > A		**Tuberous sclerosis-2** Lymphangioleiomyomatosis, somatic	Hetero			AD	PM2, PM6, PP5, PP4
96	*CACNG2*	NM_006078.3	c.437-2A > G		Mental retardation	Hetero			AD	PVS1, PM2
98	15q24 deletion (2.2Mb)									
121	**MECP2	NM_004992.3	c.455C > G	p.Pro152Arg	**{Autism susceptibility, X-linked 3}** **Mental retardation** **Rett syndrome** Encephalopathy, neonatal severe	Hetero			XR, XD	PM2, PM5, PP3, PP5
133	14q31.3-32.12 deletion					Hetero				
138	*SLC26A4	NM_000441.1	c.2168A > G	p.His723Arg	Deafness with enlarged vestibular aqueduct; Pendred syndrome	Hetero	0.0001	0.00401929	AR	PP3,PP2,PP5
	*SLC26A4	NM_000441.1	c.919-2A > G		Deafness with enlarged vestibular aqueduct; Pendred syndrome	Hetero	0.0003	0.000803859	AR	PVS1, PP5
142	**SYNGAP1	NM_006772.2	c.980T > C	p.Leu327Pro	**Mental retardation**	Hetero	–	–	AD	PM2, PP5

Seventeen participants showed likely pathogenic variants. OMIM: Online Mendelian Inheritance in Man; ExAC, population frequency from The Exome Aggregation Consortium; KRGDB, population frequency from the Korean Reference Genome Database; AD, Autosomal dominant; AR. Autosomal recessive; XD, X-linked dominant; XR, X-linked recessive; ACMG, The American College of Medical Genetics and Genomics guideline (Richards et al., 2015); PVS, Very strong evidence of pathogenicity; PM, Moderate evidence of pathogenicity; PP, Supporting evidence of pathogenicity. ^a^Inheritance of the gene described in OMIM. **BOLD: Clinical syndromes and diseases related to neurodevelopmental disorders (ASD, ID, epilepsy)**.

Importantly, pathogenic or likely pathogenic gene variants were found in approximately 43% (6/14) of participants with comorbid epilepsy. 8p23.2 duplication was the only pathogenic variant, and variations in Xp22.2p22.33 and the genes *NLGN4X, TSC2, MECP2, SYNGAP1*, were classified as likely pathogenic. Suspected genetic variants of each patient with comorbid epilepsy were shown in **Table 5**. There was no significant differences in IQ, SRS T-score, SCQ and CARS between patients with pathogenic/likely pathogenic variants and with VOUS (**Supplementary Material 3**).

**Table 5:** Genetic characteristics: genes with most suspected variants to be related to ASD with epilepsy.

No.	ACMG classification	Gene	Accession	Nucleotide	Amino acid	Diseases (OMIM)	Zygosity	Global frequency (ExAC)	Korean frequency (KRGDB)	Inheritance (OMIM)	ACMG
6	Pathogenic	8p23.2 duplication (2.2Mb)									
20	VOUS	*SCN3A*	NM_006922.3	c.5873C > G	p.Thr1958Arg	**Epilepsy, familial focal** **Epileptic encephalopathy, early infantile**	Hetero			AD	PM2
24	VOUS	*MECP2*	NM_004992.3	c.602C > T	p.Ala201Val	**{Autism susceptibility, X-linked 3}** **Mental retardation** **Rett syndrome** Encephalopathy, neonatal severe	Hemi	0.0015	0.00643087		PP3, PP5
34	VOUS	*GRIN2A*	NM_000833.4	c.3059C > G	p.Ser1020Cys	**Epilepsy, focal, with speech disorder and with or without mental retardation**	Hetero				PM2
38	VOUS	*SCN1A*	NM_006920.4	c.2556+9_2556+10insG		**Dravet syndrome** **Epilepsy, generalized, with febrile seizures plus, type 2** **Febrile seizures, familial, 3A** Migraine, familial hemiplegic	Hetero			AD	PM2
60	Likely pathogenic	Xp22.2p22.33 deletion					Hetero				
	Likely pathogenic	*NLGN4X*	NM_020742.3	Whole gene deletion		**Asperger syndrome susceptibility** **Autism susceptibility** **Mental retardation**	Hetero				
84	VOUS	*ROBO1*	NM_002941.3	c.3229C > T	p.Gln1077Ter		Hetero				PVS1, PM2
94	Likely pathogenic	*TSC2*	NM_000548.3	c.4744_4746del	p.Ile1582del	**Tuberous sclerosis-2** Lymphangioleiomyomatosis, somatic	Hetero			AD	PM2, PM4, PM6
95	Likely pathogenic	*TSC2*	NM_000548.3	C.2838-122G > A		**Tuberous sclerosis-2** Lymphangioleiomyomatosis, somatic	Hetero			AD	PM2, PM6, PP5, PP4
121	Likely pathogenic	*MECP2*	NM_004992.3	c.455C > G	p.Pro152Arg	**{Autism susceptibility, X-linked 3}** **Mental retardation** **Rett syndrome** Encephalopathy, neonatal severe	Hetero			XR, XD	PM2, PM5, PP3, PP5
122	VOUS	*ZEB2*	NM_014795.3	c.2494G > A	p.Ala832Thr	Mowat–Wilson syndrome	Hetero				PM2
142	Likely pathogenic	*SYNGAP1*	NM_006772.2	c.980T > C	p.Leu327Pro	**Mental retardation**	Hetero				PM2, PP5
143	VOUS	*LRP2*	NM_004525.2	c.5314G > A	p.Val1772Ile	Donnai**–**Barrow syndrome	Hetero				PM2, PP3
144	VOUS	*TUBGCP6*	NM_020461.3	c.4009G > A	p.Gly1337Arg	Microcephaly and chorioretionpathy	Hetero	0.00001048			PM2

Most suspected genetic variant of each ASD patients with comorbid epilepsy. OMIM, Online Mendelian Inheritance in Man; ExAC, population frequency from The Exome Aggregation Consortium; KRGDB, population frequency from the Korean Reference Genome Database; AD, Autosomal dominant; AR, Autosomal recessive; XD, X-linked dominant; XR, X-linked recessive; ACMG, The American College of Medical Genetics and Genomics guideline (Richards et al., 2015); PVS, Very strong evidence of pathogenicity; PM, Moderate evidence of pathogenicity; PP, Supporting evidence of pathogenicity. ^a^Inheritance of the gene described in OMIM. **BOLD: Clinical syndromes and diseases related to neurodevelopmental disorders (ASD, ID, epilepsy)**.

**Figure 1:**
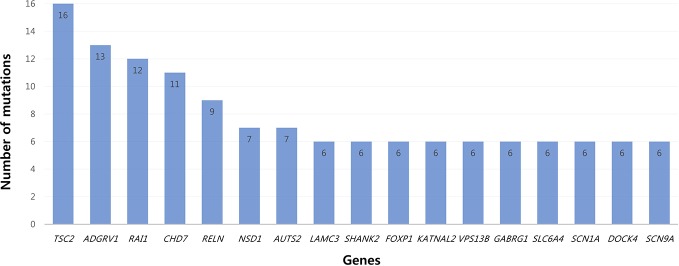
Most common genes with variants of unknown significance. Among genes with VOUS, *TSC2* (16 times) was most frequently observed. After *TSC2*, common genes appeared in the order of *ADGRV1* (13), *RAI1* (12), *CHD* (11), *RELN* (9), *NSD1/AUTS2* (7) and *LAMC3/SHANK2/FOXP1*/*KATNAL2/VPS13B/GABRG1/SLC6A4*/*SCN1A/DOCK4/SCN9A (6)*.

**Figure 2:**
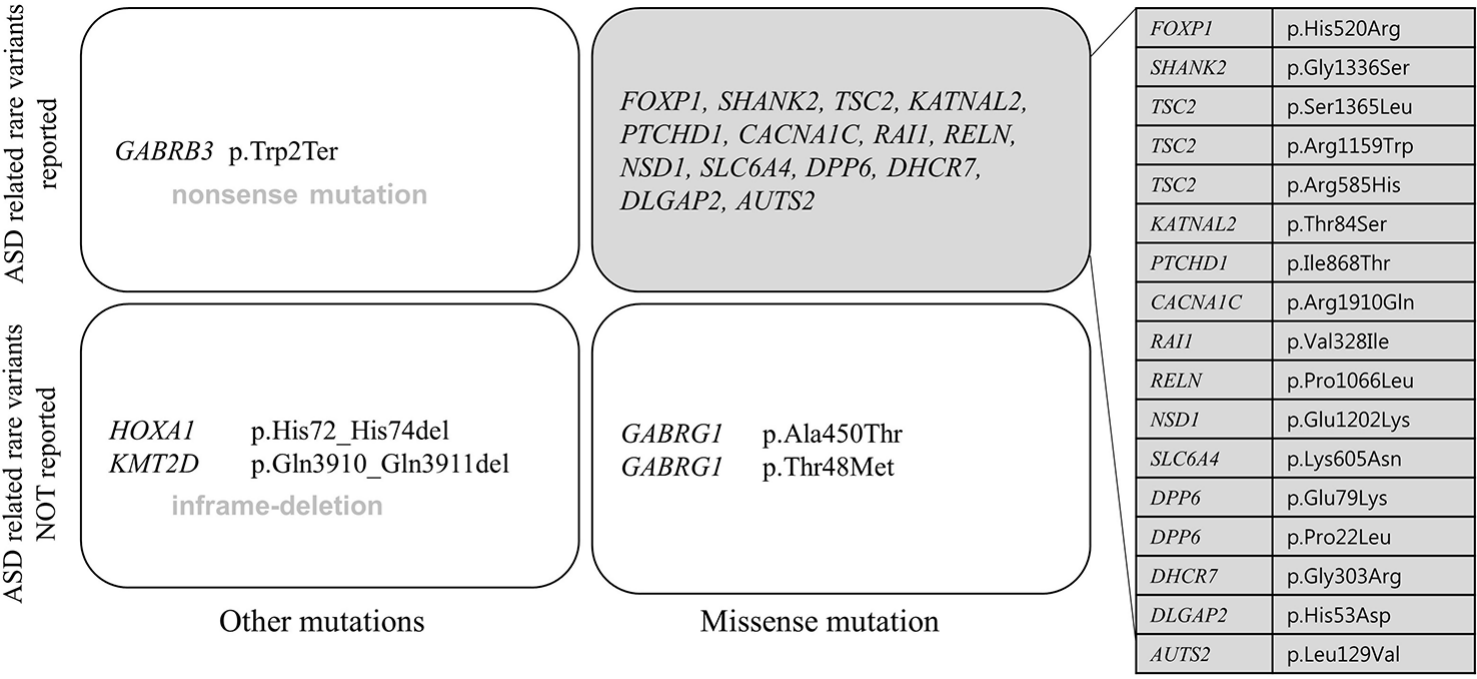
Repeatedly reported variations of unknown significance. A missense mutation (p.His520Arg) of *FOXP1* gene was most commonly found. Only *FOXP1* gene variation appeared three times in 137 patients, the rest of missense mutations were found twice each. In perspective of mutation type, one nonsense mutation (p.Trp2Ter in *GABRB3* gene), two in-frame deletions (p.Gln3910_Gln3911del in *KMT2D* gene, p.His72_His74del in *HOXA1* gene), 21 missense mutations were related to each gene. Most genes have already been reported to be related to ASD according to OMIM and SFARI database.

All patients had 0 to 37 VOUS genetic variants, 11.45 variants on average in our NGS clinical reports. There were several genes commonly observed with various variations. *TSC2, ADGRV1, RAI1, CDH7, RELN*, and *NSD1* were the most commonly reported genes with variants of unknown origin regardless of mutation types. Genes with VOUS were repeatedly identified about 1.8 times on average in our data, with standard deviation of 1.79. **Figure 1** shows the most frequently identified genes presenting VOUS in our patients without considering the variant type. More specifically, we also examined variants of unknown significance, including types of mutation and locations of the variants. As shown in **Figure 2**, an identical missense mutation in the *FOXP1* gene was found three times among 137 patients, and other missense mutations were seen twice. These results suggest that large portion of genes with VOUS were restricted to missense mutation and have already been reported as genes related to ASD according to OMIM and SFARI database.

## Discussion

Among 137 patients, only three patients showed no pathogenic, likely pathogenic variants and VOUS. This might be because patients who had severe symptoms or signs suggesting a genetic etiology in the clinical setting underwent NGS. Severe symptoms are usually related to genetic burden in ASD (Pizzo et al., 2019).

According to previous studies, diagnostic yields vary case by case (Yang et al., 2014; Tammimies et al., 2015; Rossi et al., 2017). Our yield of 17.51% may be within the predicted range, but the remarkable differences between males and females are concerning. The diagnostic yield of the female group was 28.8% which was significantly higher than that of the males. Higher SRS T-scores and CARS scores that indicate severity of autism symptoms were also significantly high in females. Though females are less prevalent in ASD (Baio et al., 2018), genetic burden and symptom severity can be higher than males. Females with ASD are known to have more genetic load than males (Lai et al., 2015), and severe clinical conditions also tend to be related with genetic variants (Lovato et al., 2019). Such reports support our results which highlight the importance of genetic evaluation in females with ASD.

### Rare Genetic Variants in Autism Spectrum Disorder

Most pathogenic variants were found in genes such as *SHANK3, PTEN, NSD1*, and the 8p23.2 duplications that have already been reported to be associated with ASD. Most pathogenic variants are related to specific neurodevelopmental syndromes. Variants in *SHANK3* can accompany Phelan**–**McDermid syndrome (Berg et al., 2018); *PTEN*, Cowden syndrome (Goffin et al., 2001); *NSD1*, Sotos syndrome (Kurotaki et al., 2002); and *RAI1*, Smith**–**Magenis syndrome (Slager et al., 2003; Laje et al., 2010). These syndromes are often reported to be related with ASD (Goffin et al., 2001; De Rubeis et al., 2014; Connolly et al., 2017). In the case of the 15q11.2–q13.2 duplication, a previously reported duplication in 15q11–13 was associated with ASD, and if variants are inherited from the father, Prader**–**Willi syndrome should also be considered (Bolton et al., 2004; Veltman et al., 2005). Autism spectrum disorder with these syndromes related genes should be monitored with caution, regarding comorbidities. However, unlike other genes, variants of *PAFAH1B1* have not been previously reported as rare variants that affect ASD development. An animal study demonstrated that mutation in the murine ortholog of this gene contributes to diminished social interaction in mice (Sudarov et al., 2013). As the gene plays a role in synaptogenesis and nervous system development (Wall et al., 2009; Sudarov et al., 2013), the possibility of ASD pathogenicity should not be neglected. The 8p23.2 duplication is described in *Rare Genetic Variants in Autism Spectrum Disorder With Comorbid Epilepsy*.

Most genes containing likely pathogenic variants were reported to be associated with neurodevelopmental disorders. The copy number variants, Xp22.2p22.33 deletion, 15q24 deletion (Adam et al., 2018), and 14q31.3–32.12 deletion (Crkvenac Gornik et al., 2019) were also reported to be related to developmental delay. Otherwise, some genes which had not been considered as ASD related genes were discovered. *ABCC2* is known to trigger Dubin**–**Johnson syndrome, which causes an increase in conjugated bilirubin levels (Keitel et al., 2003). The condition is characterized by black pigment in the liver. Mutations in *SLC26A4* have been related to Pendred syndrome, leading to sensorineuronal hearing loss (Landa et al., 2013). The participant with history of comorbid hearing loss might be due to genetic mutation in high probability, but whether this genetic mutation is also responsible for ASD development or not is unclear due to lack of evidence. *CCDC50* with a duplication in exon 11 was reported to be associated with progressive hearing loss in the Spanish group (Modamio-Høybjør et al., 2007), but to our knowledge, no reports were found to be related to *CCDC50* with neurodevelopmental disorders. Further studies are needed to understand the relationship between these genes and ASD.

### Rare Genetic Variants in Autism Spectrum Disorder With Comorbid Epilepsy

In our study, two cases with *TSC2* variation and one case with *MECP2* variation were diagnosed as tuberous sclerosis and Rett’s syndrome among ASD patients with comorbid epilepsy, upon evidence of general appearance, clinical manifestation, and brain magnetic resonance imaging. Heterozygous *SYNGAP1* gene mutations have been associated with ASD, ID, several forms of idiopathic generalized epilepsy, and delay in psychomotor development (Pinto et al., 2010; Klitten et al., 2011). Though some variants (8p23.2 duplication, Xp22.2p22.33 deletion, and *NLGN4X*) classified as likely pathogenic, the association with epilepsy has not been reported so far.

We confirmed a 2.25 Mb duplication in the short arm of chromosome 8 (8p23.2), including the *CSMD1* gene, by additional microarray examination. Though various size duplications of 8p23.2 are known to be associated with ASD and developmental delay, such as speech delay and learning difficulties (Glancy et al., 2009; Fisch et al., 2011), there was no evidence of association with epilepsy. The complement pathway is tightly controlled in the brain and disruption of microglia-specific complement receptor 3(CR3)/C3 signaling results in sustained deficits in synaptic connectivity. It is believed that deregulation of complement activity could induce aberrant synaptic elimination, which may influence susceptibility to both neurodegenerative and psychiatric disorders (Schafer et al., 2013). Although the mechanism of pathogenesis of epilepsy is not established, recent studies have reported that synaptic connectivity is associated with the development of epilepsy (Chu et al., 2010; Karoly et al., 2018). To date, there is insufficient evidence to explain the direct relationship between duplication of *CSMD1* and epilepsy. However, we suggest that the overexpression of *CSMD1* due to 8p23.2 duplication leads to abnormal synaptic connectivity and it may contribute to the occurrence of epilepsy.

It is known that deletion of chromosome Xp is associated with ID and ASD (Shinawi et al., 2009; Willemsen et al., 2012). More than 100 genes are known to be located on Xp22.2-p22.33. Males with deletions encompassing Xp22 exhibit a phenotype consistent with the loss of one or more of the genes located in this region (Melichar et al., 2007). In females, there are only a few reports that show *de novo* chromosomal deletions of Xp22 are associated with ASD and developmental delay (Thomas et al., 1999; Chocholska et al., 2006). In addition to the loss of genes located in Xp22, unfavorable X-inactivation of the intact chromosome would be another mechanism for the expressed phenotype in females (Shinawi et al., 2009). Additional tests to confirm the exact location and extent of the Xp deletion were recommended, but not performed in our case. *NLGN4X* is a gene located in the short arm of the X chromosome and is also known to be related to ASD and ID (Jamain et al., 2003; Macarov et al., 2007), but not with epilepsy. In an animal study, *Nlgn4* knock-out (KO) mice showed decreased network response and increased protein expression of synaptic proteins, such as N-methyl-D-aspartate receptor (Nmdar) subunit 1 (Nr1), and metabotropic glutamate receptor 5 (mGluR5), which are involved in synaptic plasticity and excitatory circuit rewiring (Delattre et al., 2013). Imbalance between excitatory and inhibitory synapses is one of the main hypotheses explaining the pathogenesis of epilepsy (Matsumoto and Ajmonemarsan, 1964). Furthermore, excitation/inhibition imbalances resulting from neurodevelopmental deficits have been suggested as pathogenic mechanisms for both ASD and epilepsy (Bozzi et al., 2018).

Interestingly, though estimated as VOUS, some previously reported epilepsy genes (*SCN1A, SCN3A, MECP2*, and *GRIN2A*) were also detected. Genes may have different mutation types or location, which leads to different effect sizes on ASD or epilepsy development, but they still have an important impact on disease occurrence.

### Variants of Unknown Origin

In the development of ASD, multiple loci tend to show relatively weak genotype–phenotype correlations and act additively (Persico and Napolioni, 2013). This means that common variants with low effect sizes should not be ignored considering the heterogeneity of ASD. Unfortunately until now, studies such as genome-wide association studies (GWAS), which focus on the contribution of common variants to disease, have not yield consistent results (Geschwind, 2011). According to the ACMG guideline, the VOUS variant can also have one or more evidence of pathogenicity even it was classified as VOUS. So the NGS results should be interpreted carefully, as it is possible to suggest new common variants relevant to ASD pathogenesis.

Except two genes (*ADGRV1* and *GABRG1*), 15 genes frequently classified as VOUS were also reported multiple times with rare variants in ASD (Geschwind, 2011; Persico and Napolioni, 2013; Sener et al., 2016). As shown in **Figure 2**, except one nonsense mutation and two in-frame deletions, most VOUS were missense mutations. That is, the usual types of VOUS mutation are less likely to disrupt function of gene. For this reason, even if a mutation occurs in the same gene, the effect on the development of ASD might be different depending on the mutation type.

Likewise, *ROBO1* which was identified in ASD with comorbid epilepsy was implicated in developmental dyslexia and dysfunction of language acquisition system (Hannula-Jouppi et al., 2005; Bates et al., 2011). In addition to the roles in guiding axons and the Slit/Robo signaling pathway, *ROBO1* is also involved in cellular processes such as cell migration and immune cell activation during neuroinflammatory responses (Mirakaj and Rosenberger, 2017). Recently, it has been suggested that inflammation and autoimmunity play important roles in childhood seizures and epilepsies (Korff and Dale, 2017). In our case, the patient with a nonsense mutation in *ROBO1* was diagnosed with ASD and had clinical manifestations of focal seizure. This case suggests that although a genetic variation does not satisfy the criteria of pathogenic/likely pathogenic variants, it might affect an individual’s phenotype.

Through these results, it is possible to surmise that genes with known pathogenic variants may often appear with VOUS also. As variants affect genetic functions such as synaptic and neuronal plasticity (Ben-David and Shifman, 2012), the influence on ASD would be exerted when loss of function occurred, even though the effect may vary by location and type of mutation. It is necessary not to overlook genes with VOUS if the gene has been previously reported with pathogenic variants in neurodevelopmental disorders, including ASD.

Heterogeneity has been a great challenge for developing tailored treatment of ASD as there are a large number of genes related to ASD, and loss of function differs according to each type of mutations. Through the advancement of genetic analysis technology, NGS results are being used in clinical fields, but it is still difficult to interpret and identify the clinical significance. To provide proper management to ASD individually, discrimination of the pathogenic variant among multiple variants should be achieved. Our results show that it is necessary to notice genes with VOUS although their function is not clearly defined yet. Especially in ASD presenting heterogeneous clinical manifestation and frequent comorbid disorders, results of genetic analysis should be performed with caution. The VOUS in ASD related genes involved with unclear mutation, or non-ASD related genes with clinically relevant phenotype may be of primary importance in investigating genetic data (Lovato et al., 2019). Efforts to identify the function of genes with VOUS will lead to discovering genetic pathogenesis of neurodevelopment disorder in the future.

There are several limitations to this study. First, as this study reviewed medical records retrospectively, clinical assessment could not be performed without bias. This may have influenced the statistical results of the demographic data. Second, in cases of age under 4 years, we could estimate intellectual disability only by Bayley Scales of Infant Development. Third, as our participant group mostly showed severe phenotypes (SRS T-score 86.39 on average), further studies are needed to compare differences in genetic components according to severity of ASD phenotype. Furthermore, as we analyzed the clinical NGS reports retrospectively, we could not show data from typically developing control group. To define the pathogenicity of variants of genes, comparing the result with that of normal population might be helpful. Finally, as medical records were reviewed cross-sectionally, we could not evaluate the development of comorbidities including epilepsy.

Despite the limitations mentioned above, there are several strengths in our study. First, to our knowledge, this is the first NGS study in ASD patients with or without comorbid epilepsy in Korea. As all patients are Korean, our results are not confounded by population genetic heterogeneity. Second, because NGS was carried out only in ASD patients who had severe phenotypes, comorbid disorders, or suspicious general appearance in a clinical setting, genetic variants thought to impact ASD development were able to be easily obtained. Third, we found some genes that have not previously been reported but are possibly pathogenic in ASD. Finally, by considering comorbid epilepsy, we confirmed genetic overlaps in ASD and epilepsy, even though genetic variations are currently known to be related just with either ASD or epilepsy.

In conclusion, we suggest that rare variants (pathogenic/likely pathogenic) and common variants (VOUS) are both necessary in investigating individuals’ genetic characteristics in ASD and epilepsy. Pathogenic/likely pathogenic variants might be useful in confirming genetic syndrome, predicting comorbidity, and treatment planning. The VOUS might also influence the phenotype characteristics of ASD and epilepsy, even though the evidence and possibility are not strong enough. Careful efforts in interpreting the VOUS might contribute to understand the genetic cause of ASD and epilepsy.

## Data Availability Statement

The datasets generated for this study will not be made publicly available because it includes the patient’s genetic data for clinical purpose.

## Ethics Statement

The studies involving human participants were reviewed and approved by Institutional Review Boards at Severance Hospital, Yonsei University College of Medicine. Written informed consent from the participants’ legal guardian/next of kin was not required to participate in this study in accordance with the national legislation and the institutional requirements.

## Author Contributions

Conceived and designed the experiments: JL, SH, K-AC. Performed and analyzed NGS: S-TL, S-GP, SS, JC. Analyzed data: JL, SH, S-GP, SS. Wrote the manuscript: JL, SH, S-TL, S-GP, SS, JC, K-AC.

## Conflict of Interest

The authors declare that the research was conducted in the absence of any commercial or financial relationships that could be construed as a potential conflict of interest.
