# Supplementary material for: Next-Generation Sequencing in Korean Children With Autism Spectrum Disorder and Comorbid Epilepsy
Source: Front Pharmacol. 2020 May 14;11:585. doi: 10.3389/fphar.2020.00585 (PMC7240034; doi:10.3389/fphar.2020.00585)
Supplement: Supplementary file 1 [file DataSheet_1.docx]

Supplementary material 1. Characteristics of epilepsy and Electroencephalogram (EEG) profiles in in ASD with comorbid Epilepsy

| Pt. NO. | Age | Epilepsy type | EEG | IQ | SRS | Other comorbidity | Number of AED |
| --- | --- | --- | --- | --- | --- | --- | --- |
| 6 | 5Y-10Y | Generalized | Diffuse encephalopathy | n/c | n/c | Hereditary deafness | 1 |
| 20 | 5Y-10Y | Focal with secondary generalized | Diffuse encephalopathy | 46 | 79 | none | 2 |
| 24 | 10Y-15Y | Generalized | Diffuse encephalopathy | 59 | 108 | None | 1 |
| 34 | 0Y-5Y | Generalized | Epileptiform discharge | 43 | n/c | None | 1 |
| 38 | 0Y-5Y | Repeated Febrile seizure  Infantile seizure | normal | 63 | n/c | None | 1 |
| 60 | 5Y-10Y | Focal | Diffuse encephalopathy | <39 | 98 | None | 1 |
| 84 | 0Y-5Y | Focal | Focal seizure | <47 | 107 | None | 0 |
| 94 | 5Y-10Y | Focal | Epileptiform discharge | <48 | 120 | Tuberous sclerosis | 3 |
| 95 | 0Y-5Y | Generalized | Diffuse encephalopathy with partial seizure disorder | MDI 94, PDI 74 | n/c | Tuberous sclerosis | 1 |
| 121 | 0Y-5Y | Focal with secondary generalized | Diffuse encephalopathy | MDI<50, PDI<50 | 78 | Rett’s syndrome | 3 |
| 122 | 0Y-5Y | Generalized | Normal | <40 | 70 | None | 1 |
| 142 | 5Y-10Y | Focal | Focal seizure with underlying encephalopathy | MDI<50, PDI< 50 | n/c | Lennox-  Gastaut synd. | 3 |
| 143 | 0Y-5Y | Generalized | Focal seizure | 42 | n/c | None | 1 |
| 144 | 0Y-5Y | Focal | Diffuse encephalopathy with partial seizure disorder | 45 | 68 | None | 1 |

Characteristics of epilepsy and result of electroencephalogram (EEG) were listed on the table. Every participant had symptoms of repeated seizure, and only one EEG was reported normal. Except one participant, they were using at least one antiepileptic drug (AED).

**Supplementary material 2. Clinical characteristics of ASD with pathogenic/likely pathogenic variants**

|  | **Pathogenic variants**  **(n=7)** | **Likely pathogenic variants**  **(n=17)** | ***p*-value** |
| --- | --- | --- | --- |
| **Male:Female** | 4:3 | 4:13 |  |
| **IQ** | 47.60 | 51.17 | 0.564 |
| **SRS (total T-score)** | **105** | **87** | **0.027** |
| **SCQ** | 17.67 | 18.83 | 0.751 |
| **CARS** | 30.83 | 35.26 | 0.195 |

Results of clinical and neuropsychological tests were listed on the table, and two groups(pathogenic variants group, likely pathogenic group) compared. In likely pathogenic variants group, there were much more female participants than male. Total T score of SRS were significantly higher in pathogenic variants group. There were no group differences in IQ, SCQ, and CARS score.

**Supplementary material 3. Clinical characteristics of ASD with epilepsy. Comparing pathogenic/likely pathogenic variant group and VOUS group**

|  | **Total**  **(n=14)** | **Pathogenic/likely pathogenic variants**  **(n=6)** | **VOUS**  **(n=8)** | ***p*-value** |
| --- | --- | --- | --- | --- |
| **Male:Female** | 6:8 | 1:5 | 5:3 |  |
| **IQ** | 48.13 | 43.5 | 49.67 | 0.423 |
| **SRS (total T-score)** | 94.29 | 98.67 | 91.00 | 0.638 |
| **SCQ** | 18.90 | 23.25 | 16.00 | 0.217 |
| **CARS** | 35.00 | 39.25 | 32.57 | 0.212 |

Results of clinical and neuropsychological tests in ASD with epilepsy were listed on the table. There were no group differences(Pathogenic/likely pathogenic variants group versus VOUS grou) in IQ, SRS, SCQ, and CARS scores.
